# Photographs of Distinguished Foreign Physicians, Surgeons and Specialists

**Published:** 1869-08

**Authors:** 


### Photographs of Distinguished Foreign Physicians, Surgeons and Specialists.

We have to acknowledge, with many thanks, the receipt of one hundrad card photographs, of the more distinguished foreign Physicians, Surgeons and Specialists, embracing many of the most celebrated Professors and Authors in Medicine, and the allied sciences.

Dr. Wm. H. Helm, of Sing Sing, N. Y., has collected at great cost and labor these photographs, and has at the urgent solicitation of several of his medical friends undertaken to furnish the Profession of this country with accurate copies of his collection. These copies can be had at a trifling cost compared with the originals. He has the satisfaction of stating that the copies are scarcely inferior in the slightest respect to the originals, and far more satisfactory than he had anticipated in the beginning of the enterprise. They have all been made under his immediate supervision, and in the few instances where a photograph is not fully equal to the rest of the series, it is not due to the photographer who has been employed, but to the fact that the original was not entirely satisfactory, although the best that could possibly be procured.

The subscriber wishes to express his indebtedness to Drs. Wm. H. Ford, of Philadelphia; G. J. Fisher, of Sing Sing, N.Y.; J. H. Pooley, of Yonkers, N. Y.; Wm. F. Holcombe, and Prof. Fordyce Barker, of N. Y.; for the loan of original photographs and for advice and assistance.

*Terms.*—$15.50 per set of 100. $2.00 per doz. 20 cts. each for less than 1 doz. Sent post-paid by mail.

Orders must be accompanied by the money, which should be in P. O. order, check or draft drawn to order, or registered letter.

WM. H. HELM, M. D., *Sing Sing, N. Y.*

For sale at Office of MEDICAL AND SURGICAL REPORTER, Philadelphia.
" " LINDSAY & BLACKISTON, "
" " WM. WOOD & CO., New York.

The profession are under many obligations to Dr. Helm for this effort, since many of the single photographs are really worth the price of the whole collection. Physicians who would like better acquaintance with foreign teachers and authors will avail themselves of this rare opportunity.

